# Risperidone long-acting in-situ microimplant following a brief oral risperidone lead-in in the acute inpatient management of manic episodes with psychotic symptoms in non-adherent patients with schizoaffective disorder: a retrospective, uncontrolled real-world study

**DOI:** 10.1186/s12991-026-00674-1

**Published:** 2026-05-28

**Authors:** Giovanni Barillà, Andrea Fagiolini, Debora Bussolotti, Cristina Venco, Alessandro Cuomo

**Affiliations:** 1 Department of Mental Health and Addictions, ASST Mantua, Mantua, 46100 Italy; 2 Department of Molecular Medicine, School of Medicine, University of Siena, Siena, 53100 Italy

**Keywords:** Risperidone ISM, In situ microparticles, Acute mania, Schizoaffective disorder, Bipolar disorder, Long-acting injectable antipsychotic

## Abstract

**Background:**

Nonadherence is a major cause of relapse in patients with schizophrenia, schizoaffective disorder, or bipolar disorder who have achieved remission. However, it is also a significant challenge during manic episodes. Risperidone in-situ microimplant (ISM) releases the drug early and in a sustained manner once a month, eliminating the need for daily administration, which can be difficult during a manic episode. In this study, we described symptom trajectories and tolerability of a brief oral risperidone lead-in followed off-label initiation of risperidone ISM within multimodal inpatient care in nonadherent inpatients with schizoaffective disorder experiencing a manic episode with psychotic symptoms.

**Methods:**

This retrospective, observational, single-centre study included 50 consecutive adults admitted after discontinuation/marked non-adherence and with prior response to risperidone who received ≥ 6 days of oral risperidone to confirm tolerability, then risperidone ISM (75 or 100 mg) and were followed for 6 weeks. Mania severity was assessed with the Young Mania Rating Scale (YMRS).

**Results:**

Fifty patients were included; most received concomitant mood stabilisers and benzodiazepines. Median YMRS decreased from 32 at admission (Tx) to 26 at the first injection (T0, after the oral lead-in), 8 by day 8, and remained low at day 28 (5) and day 42 (6). Activation/behavioural items improved earlier, whereas psychotic thought content improved more gradually, predominantly between days 8 and 28. Side-effect burden at weeks 4 and 6 was minimal, and no discontinuations due to adverse events occurred.

**Conclusions:**

In this exploratory uncontrolled cohort, an oral risperidone lead-in plus monthly risperidone ISM within multimodal inpatient care was associated with with rapid and sustained improvement in symptom trajectories and favourable short-term tolerability. Findings reflect multimodal inpatient care and cannot be attributed to ISM alone. Prospective, controlled studies are warranted.

**Supplementary Information:**

The online version contains supplementary material available at 10.1186/s12991-026-00674-1.

## Introduction

Schizoaffective disorder, bipolar type, is a severe mood–psychotic condition in which manic, depressive, and schizophrenia-spectrum symptoms coexist over time [1]. Longitudinal studies suggest that schizoaffective disorder occupies an intermediate position between prototypical schizophrenia and bipolar I disorder, showing a chronic or recurrent course with persistent symptoms, functional impairment and frequent need for ongoing antipsychotic treatment [2, 3].

Real-world analyses highlight a substantial burden of illness in schizophrenia and schizoaffective disorder, with high healthcare resource utilization, frequent hospitalizations and substantial direct and indirect costs [4, 5]. Across psychotic and bipolar disorders, non-adherence to antipsychotic medication is common, with many patients taking treatment on fewer than half of prescribed days and average adherence rates in the 40–60% range [6]. Even relatively brief periods of non-adherence in recent-onset schizophrenia have been associated with markedly increased risks of relapse and rehospitalization [7]. In schizoaffective disorde treatment interruptions may precipitate acute episodes with psychotic features, leading to emergency admission and further longitudinal destabilization [2–4].

Long-acting injectable (LAI) antipsychotics were developed to address some challenges of daily oral treatment by providing sustained drug delivery and reducing reliance on daily pill-taking. International guidelines recommend considering LAIs for patients with schizophrenia-spectrum disorders and bipolar disorder, particularly in those with partial adherence, frequent relapse or difficulty engaging with treatment [8]. While randomized trials in relatively adherent and stable patients have sometimes shown limited differences between LAIs and oral antipsychotics, mirror-image, cohort and registry studies in routine practice more consistently report reductions in relapse and rehospitalization after LAI initiation [9, 10]. Network meta-analytic evidence also supports the role of both oral and long-acting antipsychotics for relapse prevention across schizophrenia-spectrum disorders [11].

Risperidone, a second-generation antipsychotic, is widely used across the schizophrenia–bipolar–schizoaffective spectrum. Randomized and open-label studies have demonstrated antimanic efficacy of risperidone, including long-acting formulations, in bipolar I disorder and have suggested benefits in schizoaffective disorder, particularly as maintenance therapy in patients with frequent relapses or poor adherence [12–16]. These data support the broader concept that risperidone-based LAIs can provide both antipsychotic and mood-stabilizing effects in mood–psychotic disorders.

Risperidone in situ microparticles (risperidone ISM) is a once-monthly LAI formulation that employs an in situ microimplant technology to achieve a biphasic pattern of drug release. After intramuscular injection, a fraction of the dose is rapidly released, while the remainder forms a biodegradable depot that provides controlled risperidone delivery over the 4-week dosing interval [17, 18]. Phase II–III trials and extension studies in schizophrenia have shown that risperidone ISM has been associated with rapid improvements in psychotic symptoms, maintains therapeutic exposure over the dosing interval and has a tolerability profile broadly comparable to oral risperidone [17, 18]. However, current evidence for risperidone ISM is largely confined to schizophrenia, and— to our knowledge—no published clinical studies have specifically evaluated this formulation in bipolar I disorder or in schizoaffective disorder, bipolar type, particularly in the context of acute manic episodes with psychotic symptoms and recent antipsychotic non-adherence.

Given (i) the established antimanic efficacy of oral and long-acting risperidone in bipolar and schizoaffective populations [12–15], (ii) the central role of LAIs in the management of non-adherent patients [8, 10], and (iii) the pharmacokinetic profile of risperidone ISM—combining early release with once-monthly dosing [17, 18]—a systematic real-world evaluation of risperidone ISM in schizoaffective mania appears clinically warranted. This retrospective study was therefore undertaken to characterize the short-term clinical course and tolerability of a pragmatic “oral risperidone lead-in plus monthly risperidone ISM” (off-label) strategy in a consecutive series of non-adherent inpatients with schizoaffective disorder, bipolar type, hospitalized for an acute manic episode with psychotic features and a documented previous clinical response to risperidone.

## Methods

### Ethical approval and informed consent statement

The study protocol (acronym “REALSAD”) was approved by the Territorial Ethics Committee CET Lombardia 4 (promoter: ASST Mantova; protocol code CET 140/24). All procedures were conducted in accordance with the Declaration of Helsinki and Good Clinical Practice guidelines. After clinical stabilisation during the index hospitalisation and/or at routine follow-up visits, all pa-tients provided written informed consent for the collection and use of anonymised clinical data for research purposes, as required by the local Ethics Committee. Decisional capacity was assessed clinically by the treating psychiatrist at the time consent was obtained; therefore, proxy consent was not required in this cohort.

### Study design and setting

This was a retrospective, observational, single-centre study conducted in the Psychiatry Unit Mantova 1 (S.C. Psichiatria, ASST Mantova, Mantova, Italy). Electronic medical records of consecutive in-patients with schizoaffective disorder (SAD) were reviewed. Patients were selected according to the formal diagnosis and documentation available in the clinical charts, adhering to DSM-5-TR criteria for schizoaffective disorder, bipolar type, currently manic episode with psychotic symptoms [1]. The study included admissions between January 2024 and November 2025.

The study followed the STROBE recommendations for observational studies [19]. The completed STROBE checklist is provided as an *Additional file 1 - STROBE Checklist*. Clinical management (choice of antipsychotic, adjunctive mood stabilisers or benzodiazepines) was entirely naturalistic and based on the treating psychiatrist’s judgement. No study-specific procedures were introduced; all measures derived from routine clinical care and retrospective chart review.

#### Participants – inclusion criteria

Patients were eligible for inclusion if they:

1. Were aged ≥ 18 years and admitted to the adult psychiatric inpatient unit.
2. Had a primary diagnosis of schizoaffective disorder, bipolar type, in a current manic episode with psychotic features according to DSM-5-TR [1].
3. Had clinical relapse temporally associated with discontinuation or marked non-adherence to ongoing antipsychotic treatment, defined as taking < 50% of prescribed doses during the month preceding admission, as documented in the medical record. Estimates of adherence were based on routine clinical documentation available in the medical record (e.g., clinical history, family report, and collateral information when available).
4. Received risperidone ISM (in-situ microimplant) during the index hospitalisation as part of routine clinical care, with at least one injection recorded.
5. Had a documented history of previous clinical response to risperidone (oral or depot formulation), reported in previous treatment episodes. Documented prior response was defined as clinician-documented improvement of relevant manic and/or psychotic symptoms during a previous treatment episode with oral or long-acting risperidone, as reported in the clinical record (e.g., discharge summaries and outpatient/inpatient notes). Given the retrospective nature of the study, this ascertainment was based on routine documentation and did not require standardized symptom ratings.
6. Had Young Mania Rating Scale (YMRS) and Clinical Global Impression (CGI) scores available at admission (Tx) and at least one subsequent post-injection time point.

#### Exclusion criteria

Patients were excluded if they:

1. Had an alternative primary diagnosis (e.g. bipolar disorder without psychosis, schizophrenia, schizophreniform disorder) or if the current manic/psychotic presentation was judged to be primarily substance-induced (i.e. attributable to acute intoxication or withdrawal from alcohol or illicit substances).
2. Had incomplete or inconsistent clinical documentation, precluding reliable reconstruction of treatment adherence estimation, treatment timing or rating-scale trajectories.
3. Were pregnant or breastfeeding at the time of hospitalisation, or had a documented contraindication to risperidone according to the summary of product characteristics (e.g. severe, unstable cardiovascular disease, known hypersensitivity to risperidone or excipients).

Included patients were diagnosed in routine clinical practice by board-certified psychiatrists using DSM-5-TR criteria and confirmed by the Mini International Neuropsychiatric Interview [20]. In this retrospective cohort, MINI confirmation refers to structured diagnostic information documented in the clinical record from the index admission when available, or from prior assessments included in the patient history when already available. When MINI documentation was not already available in the clinical history, the MINI was administered during the index hospitalization as part of routine clinical assessment and documented in the medical record. In the following text we refer to “SAD mania” to indicate manic episodes occurring within schizoaffective disorder, bipolar type.

##### Data sources and measurements

Data were extracted from electronic charts and structured case-report forms and included:

- Demographic and clinical variables: age, sex, body mass index (BMI), duration of diagnosed illness, number of previous mood episodes, history of substance abuse (none, alcohol, cannabis), cluster-B personality disorder, medical comorbidities, and concomitant psychotropic medications (lithium, valproate, haloperidol, other antipsychotics, benzodiazepines). Because of the retrospective design and non-uniform charting of day-by-day prescriptions (including PRN administrations), longitudinal dose data for concomitant medications were not consistently retrievable and were therefore not analysed quantitatively.
- Risperidone ISM exposure: dose of the first injection at T0 (75 or 100 mg), dose at T4 (100 mg, 75 mg, stopped depot, or switched to another long-acting antipsychotic), and timing of each injection.

*Assessment time points*

Rating scales were collected as part of routine care at the following time points:

- Tx (admission) – baseline, before risperidone ISM; demographic and clinical data; YMRS and CGI-BP.
- T0 (injection 1) – day of first risperidone ISM injection; YMRS and CGI-BP.
- T1 (24 h) – approximately 24 h after T0; YMRS and CGI-BP.
- T2 (48 h) – approximately 48 h after T0; YMRS and CGI-BP.
- T3 (day 8 ± 1 day) – early post-acute phase; YMRS and CGI-BP.
- T4 (day 28 ± 2 days; injection 2) – consolidation phase and second risperidone ISM injection; YMRS, CGI-BP and Glasgow Antipsychotic Side-Effect Scale (GASS).
- T5 (day 42 ± 2 days) – follow-up visit; YMRS, CGI-BP and GASS.

YMRS, CGI-BP ratings were completed and, including GASS ratings, recorded in routine clinical practice by the treating psychiatrists during the index hospitalization and follow-up visits; no independent raters were used. No formal inter-rater reliability exercises were conducted for the purposes of this retrospective study.

*Symptom rating scales*

- **Young Mania Rating Scale (YMRS)**
- Mania severity was assessed with the 11-item Young Mania Rating Scale (YMRS; total score 0–60) [21]. In addition, we planned a priori to describe the temporal course of individual YMRS items across all time points. Analyses of single-item trajectories were considered exploratory and primarily descriptive.
- **Clinical Global Impression for Bipolar Disorder (CGI-BP)**
- Global illness severity was rated at each time point using the Clinical Global Impression for Bipolar Disorder–Severity scale (CGI-BP-S; 1 = normal, not at all ill; 7 = among the most extremely ill patients), anchored to overall bipolar illness (overall anchor) [22]. This global anchor was selected to complement the symptom-specific YMRS by capturing the treating clinician’s overall impression of illness severity across the acute manic/psychotic presentation.

*Tolerability and adverse events*

- **Glasgow Antipsychotic Side-Effect Scale (GASS).** Tolerability was evaluated at T4 (day 28) and T5 (day 42) using an Italian-language, patient self-report version of the Glasgow Antipsychotic Side-Effect Scale (GASS; 22 scored items, total score 0–63), capturing nine domains: sedation/CNS, cardiovascular, extrapyramidal, anticholinergic, gastrointestinal, genitourinary, prolactinaemic/sexual, diabetes-screening items, and weight gain [23]. The GASS assesses the frequency of common antipsychotic side effects over the previous week (items 1–20) and includes two screening items referring to the last three months (sex-specific menstrual/erectile item and weight gain) [23]. In routine clinical practice within our unit, the GASS was used primarily to capture patient-perceived burden; therefore, the tolerability analyses in this study were intentionally focused on symptoms that patients rated as clinically distressing. In addition to the standard frequency scoring used to derive the total and domain scores, the GASS includes a patient-rated distress field for each endorsed symptom (either a “distressing” tick-box or a 1–10 distress rating), which is not included in the total score but is intended to inform the clinician of the patient’s views and condition. For the purpose of this study, we operationalised “clinically distressing side effects” a priori as symptoms with non-zero frequency on the GASS and associated with meaningful patient-perceived burden (distress ≥ 5/10 when the numeric distress rating was used, or “distressing” tick-box when that format was used), subsequently reviewed by the treating clinician as clinically relevant. Accordingly, non-distressing symptoms (i.e., endorsed but rated as non-distressing) were not included in the tolerability prevalence summaries presented in the Results, which should therefore be interpreted as reflecting the burden of *distressing side effects* rather than the overall frequency of all side effects. This approach may underestimate overall side-effect occurrence but was chosen to emphasise clinically meaningful patient burden.

Psychometric properties of the Italian translation have been reported in an Italian sample of patients with schizophrenia and bipolar spectrum disorders, showing good internal consistency [23]. 

This approach may underestimate overall side-effect occurrence but was chosen to emphasise clinically meaningful patient burden.The GASS was self-completed by patients at weeks 4 and 6 when clinically stable and able to understand the questionnaire; treating clinicians were available to clarify item meaning if needed, without suggesting responses. Forms were reviewed for completeness as part of routine visit documentation; no item-level imputation was performed beyond standard scoring.

- Severe adverse events (AEs)

All adverse events documented in the charts during hospitalisation and follow-up (e.g. extrapyramidal symptoms, sedation, metabolic changes, prolactin-related AEs) were extracted and qualitatively summarised.

### Risperidone ISM treatment protocol

Risperidone ISM (in-situ microimplant) was prescribed according to the approved summary of product characteristics and local clinical practice. In brief, oral risperidone was initiated or optimised during the first days of admission and maintained for at least 6 consecutive days to confirm both clinical stabilisation and tolerability, in line with the prescribing information. The first intramuscular risperidone ISM injection at T0 (75 mg or 100 mg) was then administered on the first available day after completion of this ≥ 6-day tolerability period. In keeping with dose-conversion recommendations, 100 mg ISM was generally used in patients who had been receiving ≥ 4 mg/day of oral risperidone, whereas 75 mg ISM was selected in those stabilised on 3 mg/day, with adjustments left to the discretion of the treating psychiatrist based on overall clinical status and comorbidities.

The choice of 75 vs. 100 mg at T0 was at the discretion of the treating clinician, based on prior exposure, severity of the manic episode, comorbidities and concomitant medications. As per the label, risperidone ISM provides an early release of risperidone without requiring oral supplementation; no systematic oral overlap was maintained beyond the acute titration period.

In the present cohort, the oral risperidone dose immediately preceding the first ISM injection at T0 was 3 mg/day in all patients initiated on 75 mg ISM. Among patients initiated on 100 mg ISM, the oral dose at T0 was 4 mg/day in the majority, whereas five patients with the most severe manic/psychotic presentation were titrated to 6 mg/day prior to T0. As per prescribing information, oral supplementation was not routinely required and oral risperidone was not continued after T0. Adjunct oral antipsychotics were used only transiently in a small subset with persistent psychotic symptoms and were discontinued by day 8 post-injection. Benzodiazepines were progressively tapered after the acute phase during follow-up, and mood stabiliser prescriptions (lithium and/or valproate), when present, were generally maintained without systematic escalation from T0.

At T4 (day 28 ± 2 days) patients were re-evaluated. In those who continued risperidone ISM, a second injection was administered at either 100 mg or 75 mg. In a minority of cases the depot was stopped or switched to another long-acting antipsychotic, again according to clinical judgement.

#### Variables and outcomes

The primary outcome was the change in YMRS total score across subsequent time points, with particular focus on the short-term antimanic effect (Tx to T2 and T3) and the maintenance of improvement at T4 and T5. For inferential purposes, the primary prespecified contrast for YMRS total score was the change from Tx (admission) to T3 (day 8 ± 1), reflecting short-term improvement during the acute pathway. Key secondary contrasts examined maintenance of improvement at T4 (day 28) and T5 (day 42). Analyses of YMRS single items were considered exploratory.

Secondary endpoints included:

- Change in CGI-BP score across the same time points.
- Trajectories of individual YMRS items, and, specifically, core YMRS items (items 5, 8, 9), describing the temporal profile of irritability, aggressive behaviour and psychotic thought content.
- The proportion of patients achieving clinical response (defined as ≥ 50% reduction in YMRS total score from admission) and complete symptomatic remission (YMRS total score < 8).

Tolerability outcomes included:

- Prevalence of clinically distressing side effects by domain at T4 and T5 (as defined a priori using the GASS distress field and clinical review),
- Distribution of distress-filtered GASS burden at T4 and T5 (see Statistical analysis),
- Frequency and nature of clinically recorded serious side effects and severe adverse events.

### Statistical analysis

Continuous variables are presented as mean ± standard deviation (SD) or median and interquartile range [IQR], according to their distribution. Categorical variables are reported as absolute and relative frequencies (n, %). Normality of continuous variables was assessed visually (histograms, Q–Q plots) and with the Shapiro–Wilk test.

Because YMRS and CGI-BP scores did not consistently meet normality assumptions, overall changes across time (Tx, T0, T1, T2, T3, T4, T5) were first evaluated using the Friedman test for repeated measures. When overall tests were significant, pairwise comparisons between baseline (Tx) and each post-baseline time point were performed using the Wilcoxon signed-rank test. To control for multiple testing, Holm–Bonferroni correction was applied to the family of comparisons. The primary inferential family was defined a priori as the set of baseline comparisons (Tx vs. each post-baseline time point) for YMRS total score and CGI-BP.

For YMRS single items, analogous Friedman and Wilcoxon procedures were used to assess changes over time. Multiplicity control was applied within prespecified families of comparisons. For YMRS total score and CGI-BP, Holm–Bonferroni adjustment was applied to the set of baseline comparisons (Tx vs. each post-baseline timepoint). For YMRS single items, Holm–Bonferroni adjustment was applied within each item across the baseline comparisons (Tx vs. each post-baseline timepoint). All item-level inferential results were considered exploratory and interpreted with caution given multiple comparisons.

In exploratory post-hoc analyses, additional Wilcoxon signed-rank tests were used to compare all pairs of post-baseline time points within each measure. These post-baseline comparisons were considered exploratory, were not used to support primary conclusions, and are not emphasized in the main text.

In addition, linear mixed-effects models for repeated measures were fitted for YMRS total and CGI-BP severity scores, with time (Tx, T0, T1, T2, T3, T4, T5) entered as a categorical fixed effect and a random intercept for patient. Least-squares means (estimated marginal means) were derived for each timepoint, and prespecified pairwise contrasts were tested for selected time comparisons (YMRS: Tx vs. T0, T0 vs. T1, T0 vs. T2, T2 vs. T3, T3 vs. T4, T4 vs. T5; CGI-BP: Tx vs. T0, T0 vs. T2, T2 vs. T3, T3 vs. T4) using Wald statistics and 95% confidence intervals. For YMRS and CGI-BP contrasts, p-values were adjusted for multiplicity using the Holm–Bonferroni method. In sensitivity analyses, the YMRS and CGI-BP mixed models were additionally adjusted for potential confounders, including age, sex, body mass index and concurrent psychotropic medications (e.g. lithium, valproate). Covariate adjustment was limited to variables reliably available in the retrospective dataset; time-varying dosing of benzodiazepines and other short-term co-interventions could not be modelled comprehensively.

Nonparametric repeated-measures tests (Friedman/Wilcoxon) were considered the primary inferential approach given distributional assumptions. Linear mixed-effects models were additionally fitted as complementary analyses to estimate timepoint means and 95% confidence intervals under a repeated-measures framework. Although CGI-BP is an ordinal scale, mixed-model results were interpreted in conjunction with the nonparametric analyses, which showed consistent conclusions across time. Mixed-effects models were therefore intended as complementary analyses to provide estimated timepoint means and confidence intervals.

There were no missing YMRS/CGI-BP repeated measures at prespecified time points; analyses were conducted on complete cases.

GASS total scores at T4 and T5 were summarised descriptively. Given that the tolerability focus was on clinically distressing symptoms, analyses were based on a distress-filtered approach: for each GASS item, the frequency score was retained only if the symptom met the predefined “clinically distressing” criterion (distress ≥ 5/10 or “distressing” tick-box, plus clinical review); otherwise the item was coded as 0 for the purpose of distress-burden summaries. Domain prevalences were described as the percentage of patients with ≥ 1 clinically distressing symptom within each domain. These summaries do not represent the overall frequency of all antipsychotic side effects, but rather the burden of distressing side effects as perceived by patients. All GASS analyses were descriptive.

All tests were two-sided. Statistical significance was set at *p* < 0.05, with Bonferroni-type corrections applied where indicated. Statistical analyses were performed using SAS 9.4 (SAS Institute Inc., Cary, NC, USA).

## Results

### Cohort description

During the study period, 66 hospitalised patients with a diagnosis of schizoaffective disorder, bipolar type, manic episode with psychotic features were screened. Of these, 9 patients were excluded because clinical documentation was incomplete or inconsistent, preventing a reliable reconstruction of treatment timing or rating-scale trajectories, and 7 patients were excluded because the acute presentation was considered predominantly substance-induced, with manic and psychotic symptoms occurring in the context of marked alcohol or illicit-substance intoxication or withdrawal, rather than a primary schizoaffective manic episode.

The final cohort therefore consisted of 50 in-patients who fulfilled all inclusion and exclusion criteria. All 50 patients completed all seven planned clinical assessments (Tx to T5), and GASS forms were available for all patients at both T4 and T5. Sociodemographic and clinical characteristics are summarised in Table 1.

**Table 1:** Sociodemographic and clinical characteristics; concomitant medications at baseline

Characteristic	Value
Sample size	*N* = 50
Age, years	37.5 [28.2–48.0] (mean 39.8 ± 14.0)
BMI, kg/m²	24.0 [21.0–31.0] (mean 26.2 ± 6.9)
Duration of diagnosed illness, years	7.5 [4.0–15.0] (mean 11.3 ± 10.5)
Number of mood episodes	3.0 [2.0–4.0] (mean 3.6 ± 2.6)
Sex, male	35 (70.0%)
Sex, female	15 (30.0%)
Substance abuse: none	32 (64.0%)
Substance abuse: alcohol	10 (20.0%)
Substance abuse: cannabis	8 (16.0%)
Personality disorder, cluster B	7 (14.0%)
Medical comorbidity	17 (34.0%)
Risperidone ISM first injection (T0)	75 mg: 9 (18.0%); 100 mg: 41 (82.0%)
Risperidone ISM second injection (T4) – dose	75 mg: 7 (14.0%); 100 mg: 39 (78.0%)
Risperidone ISM second injection (T4) – change	Stopped depot: 2 (4.0%); Switched depot: 2 (4.0%)
*Concomitant medications at baseline*	
*Psychotropic drugs*	*Patients*,* n (%)*
Lithium	14 (28.0%)
Valproate	33 (66.0%)
Haloperidol	2 (4.0%)
Other antipsychotics	9 (18.0%)
Benzodiazepines	36 (72.0%)

The median age was 37.5 years [IQR 28.2–48.0], and 35 patients (70.0%) were male.

At baseline, lithium was prescribed in 14 patients (28.0%), valproate in 33 (66.0%), haloperidol in 2 (4.0%), other antipsychotics in 9 (18.0%), and benzodiazepines in 36 (72.0%) (Table 1).

With regard to risperidone ISM exposure, the first injection at T0 was 75 mg in 9 patients (18.0%) and 100 mg in 41 (82.0%), consistent with the preceding oral dose (75 mg in patients on 3 mg/day oral risperidone and 100 mg in those on ≥ 4 mg/day). Specifically, all patients receiving 75 mg ISM (*n* = 9) had been stabilised on 3 mg/day oral risperidone prior to T0. Among those receiving 100 mg ISM (*n* = 41), most were stabilised on 4 mg/day, while five patients with the most severe manic/psychotic presentation were titrated to 6 mg/day prior to T0. No patient continued oral risperidone after T0. Adjunct oral antipsychotics were used only transiently in a small subset of patients with persistent psychotic symptoms and were discontinued by day 8 post-injection. Benzodiazepines were tapered during follow-up and discontinued by day 28 in 34 patients; the remaining patients continued low-dose lorazepam mainly at bedtime (most commonly 1–2.5 mg, with PRN doses when needed, as per routine care documentation). Lithium and valproate prescriptions, when present, were continued from T0 with no documented discontinuations; however, given the retrospective nature of the dataset, time-varying dosing adjustments could not be systematically extracted.

At T4 (day 28 ± 2 days), a second injection was administered in the majority of cases (100 mg in 39/50, 78.0%; 75 mg in 7/50, 14.0%), whereas the depot was stopped in 2 patients (4.0%) and switched to another long-acting antipsychotic in 2 (4.0%).

At admission (Tx), the cohort presented with moderate-to-severe mania and marked psychotic symptoms. The median YMRS total score was 32 [29–36], and the CGI-BP score was 6 [5–6], indicating patients who were severely ill (Table 2).

**Table 2:** CGI-BP, YMRS total and YMRS single items across time points

Measure	Tx	T0	T1	T2	T3	T4	T5	*p* (Friedman)
CGI-BP score	6 [5–6]	5 [4–6]⁎	4 [4–5]⁎ᵃ	4 [4–5]⁎ᵃ^,^ᵇ	4 [3–4]⁎ᵃ^,^ᵇ^,^ᶜ	3 [2–4]⁎ᵃ^,^ᵇ^,^ᶜ^,^ᵈ	3 [2–3]⁎ᵃ^,^ᵇ^,^ᶜ^,^ᵈ^,^ᵉ	< 0.0001
YMRS total score	32 [29–36]	26 [21–30]⁎	20 [15–30]⁎ᵃ	17 [10–27]⁎ᵃ^,^ᵇ	8 [6–11]⁎ᵃ^,^ᵇ^,^ᶜ	5 [4–8]⁎ᵃ^,^ᵇ^,^ᶜ^,^ᵈ	6 [4–8]⁎ᵃ^,^ᵇ^,^ᶜ^,^ᵈ^,^ᵉ	< 0.0001
YMRS item 1 – Elevated mood	2 [2–2]	2 [1–2]⁎	2 [1–2]⁎ᵃ	1 [0–2]⁎ᵃ^,^ᵇ	0 [0–1]⁎ᵃ^,^ᵇ^,^ᶜ	0 [0–1]⁎ᵃ^,^ᵇ^,^ᶜ	0 [0–1]⁎ᵃ^,^ᵇ^,^ᶜ	< 0.0001
YMRS item 2 – Increased activity/energy	2 [2–3]	2 [2–2]⁎	2 [1–2]⁎ᵃ	1 [0–2]⁎ᵃ	0 [0–1]⁎ᵃ^,^ᵇ^,^ᶜ	0 [0–1]⁎ᵃ^,^ᵇ^,^ᶜ^,^ᵈ	0 [0–1]⁎ᵃ^,^ᵇ^,^ᶜ	< 0.0001
YMRS item 3 – Increased sexual interest	2 [1–2]	1 [0–2]⁎	1 [0–2]⁎	1 [0–2]⁎	0 [0–1]⁎ᵃ^,^ᵇ^,^ᶜ	0 [0–1]⁎ᵃ^,^ᵇ^,^ᶜ	0 [0–1]⁎ᵃ^,^ᵇ^,^ᶜ	< 0.0001
YMRS item 4 – Reduced sleep	2 [2–3]	2 [1–2]⁎	2 [1–2]⁎ᵃ	1 [0–2]⁎ᵃ^,^ᵇ	0 [0–1]⁎ᵃ^,^ᵇ^,^ᶜ	0 [0–1]⁎ᵃ^,^ᵇ^,^ᶜ^,^ᵈ	0 [0–1]⁎ᵃ^,^ᵇ^,^ᶜ	< 0.0001
YMRS item 5 – Irritability	5 [4–5]	4 [3–5]⁎	2 [2–3]⁎ᵃ	2 [2–3]⁎ᵃ	1 [1–2]⁎ᵃ^,^ᵇ^,^ᶜ	1 [0–1]⁎ᵃ^,^ᵇ^,^ᶜ^,^ᵈ	1 [0–1]⁎ᵃ^,^ᵇ^,^ᶜ^,^ᵈ	< 0.0001
YMRS item 6 – Speech (rate and amount)	5 [3–5]	3 [3–5]⁎	2 [2–4]⁎ᵃ	2 [1–4]⁎ᵃ^,^ᵇ	1 [1–2]⁎ᵃ^,^ᵇ^,^ᶜ	1 [0–1]⁎ᵃ^,^ᵇ^,^ᶜ^,^ᵈ	1 [0–1]⁎ᵃ^,^ᵇ^,^ᶜ^,^ᵈ	< 0.0001
YMRS item 7 – Language/thought disorder	3 [3–5]	3 [2–5]	3 [2–5]⁎ᵃ	3 [2–4]⁎ᵃ	1 [1–2]⁎ᵃ^,^ᵇ^,^ᶜ	1 [1–1]⁎ᵃ^,^ᵇ^,^ᶜ^,^ᵈ	1 [0–2]⁎ᵃ^,^ᵇ^,^ᶜ^,^ᵈ	< 0.0001
YMRS item 8 – Thought content	5 [5–5]	5 [4–5]	5 [4–5]⁎	4 [3–5]⁎ᵃ^,^ᵇ	2 [1–3]⁎ᵃ^,^ᵇ^,^ᶜ	2 [1–2]⁎ᵃ^,^ᵇ^,^ᶜ^,^ᵈ	2 [1–2]⁎ᵃ^,^ᵇ^,^ᶜ^,^ᵈ	< 0.0001
YMRS item 9 – Disruptive/aggressive behaviour	2 [2–5]	2 [1–2]⁎	2 [1–2]⁎ᵃ	1 [0–2]⁎ᵃ	0 [0–1]⁎ᵃ^,^ᵇ^,^ᶜ	0 [0–0]⁎ᵃ^,^ᵇ^,^ᶜ	0 [0–0]⁎ᵃ^,^ᵇ^,^ᶜ	< 0.0001
YMRS item 10 – Appearance	2 [2–2]	2 [1–2]⁎	2 [1–2]⁎	2 [1–2]⁎ᵃ	1 [0–1]⁎ᵃ^,^ᵇ^,^ᶜ	0 [0–1]⁎ᵃ^,^ᵇ^,^ᶜ^,^ᵈ	0 [0–1]⁎ᵃ^,^ᵇ^,^ᶜ^,^ᵈ	< 0.0001
YMRS item 11 – Insight	3 [2–3]	2 [2–3]⁎	2 [2–3]⁎	2 [2–2]⁎ᵃ	1 [1–2]⁎ᵃ^,^ᵇ^,^ᶜ	1 [1–1]⁎ᵃ^,^ᵇ^,^ᶜ^,^ᵈ	1 [1–2]⁎ᵃ^,^ᵇ^,^ᶜ	< 0.0001

Values are median [IQR]. ⁎p < 0.05 vs Tx (primary baseline family; Holm–Bonferroni adjusted); ᵃp < 0.05 vs T0; ᵇp < 0.05 vs T1; ᶜp < 0.05 vs T2; ᵈp < 0.05 vs T3; ᵉp < 0.05 vs T4 (post-baseline comparisons exploratory) (Wilcoxon signed-rank tests with Holm–Bonferroni correction for multiple pairwise comparisons)

### YMRS total score

**Fig. 1:**
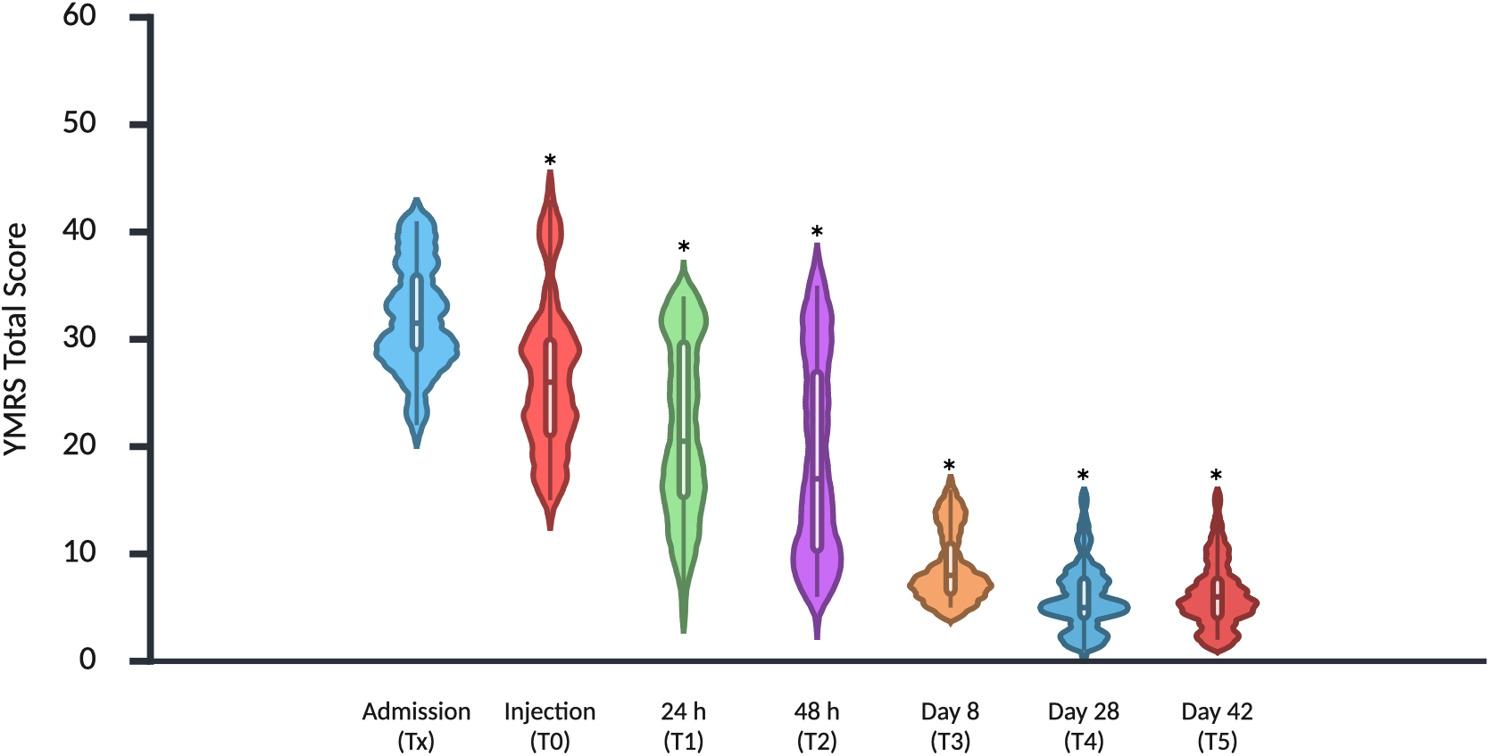
YMRS total score over time. Violin plots (with embedded boxplots) summarising the distribution of Young Mania Rating Scale (YMRS; total score range 0–60) scores across assessment time points: admission/baseline (Tx), day of first risperidone ISM injection (T0; following ≥6 consecutive days of oral risperidone lead-in and inpatient stabilisation), 24 h (T1), 48 h (T2), day 8 ± 1 (T3), day 28 ± 2 (T4; second injection), and day 42 ± 2 (T5). Embedded boxplots depict the median and interquartile range, with whiskers indicating dispersion. * indicates statistically significant reductions versus baseline (Tx) using Wilcoxon signed-rank tests with Holm–Bonferroni correction (p<0.05)

A marked and progressive improvement in YMRS total score was observed over the 6-week observation period (Friedman *p* < 0.0001; Table 2; Fig. 1). The median score decreased from 32 [29–36] at Tx to 26 [21–30] at T0, 20 [15–30] at T1 (24 h), and 17 [10–27] at T2 (48 h). Compared with Tx, YMRS total scores were significantly lower at all subsequent time points (T0–T5; all Wilcoxon *p* < 0.05 after Holm–Bonferroni correction).

**Pre-injection phase (Tx to T0).** During the oral risperidone lead-in and standard inpatient management, YMRS total score decreased from 32 [29–36] at admission (Tx) to 26 [21–30] at T0 (first ISM injection). The initial reduction between Tx and T0 occurred during the ≥ 6-day oral risperidone lead-in, while using concomitant medications (valproate, lithium, other APs and benzodiazepines) and standard inpatient management, before administration of the first risperidone ISM injection at T0.

**Post-injection phase (T0 to follow-up).** After the first ISM injection, YMRS further decreased to 20 [15–30] at 24 h (T1), 17 [10–27] at 48 h (T2), 8 [6–11] by day 8 (T3), and remained low at day 28 (T4; 5 [4–8]) and day 42 (T5; 6 [4–8]) (Table 2).

In line with the prespecified primary contrast (Tx to T3), the median YMRS decreased from 32 to 8 by day 8.

These trajectories are graphically illustrated in Fig. 1, where both medians and dispersion clearly show a rapid symptom reduction under the acute inpatient pathway followed by stabilisation and maintenance of low YMRS scores through week 6.

Mixed-effects models were consistent with rapid and clinically relevant LS-mean reductions in YMRS from admission through day 8, with all prespecified contrasts from Tx to T3 reaching statistical significance, followed by more modest improvement between day 8 and day 28 and stabilisation thereafter (see Additional file 2 : Supplementary - Table S1)*.*

Exploratory pairwise analyses further showed that YMRS total scores not only improved significantly from Tx to each post-baseline time point, but also declined stepwise from T0 to subsequent visits up to day 8, with most comparisons between T0 and T1–T3 reaching statistical significance (Table 2).

Using the predefined YMRS-based criteria, all patients met the ≥ 50% response threshold from day 8 (T3) onwards. Symptomatic remission (YMRS < 8) was achieved by 24/50 patients (48.0%) at T3 and by 37/50 (74.0%) at both T4 and T5.

Exploratory analyses of baseline correlates of day-8 remission and symptom change are reported in Additional file 2 - Supplementary (Table S3).

#### CGI-BP score

**Fig. 2:**
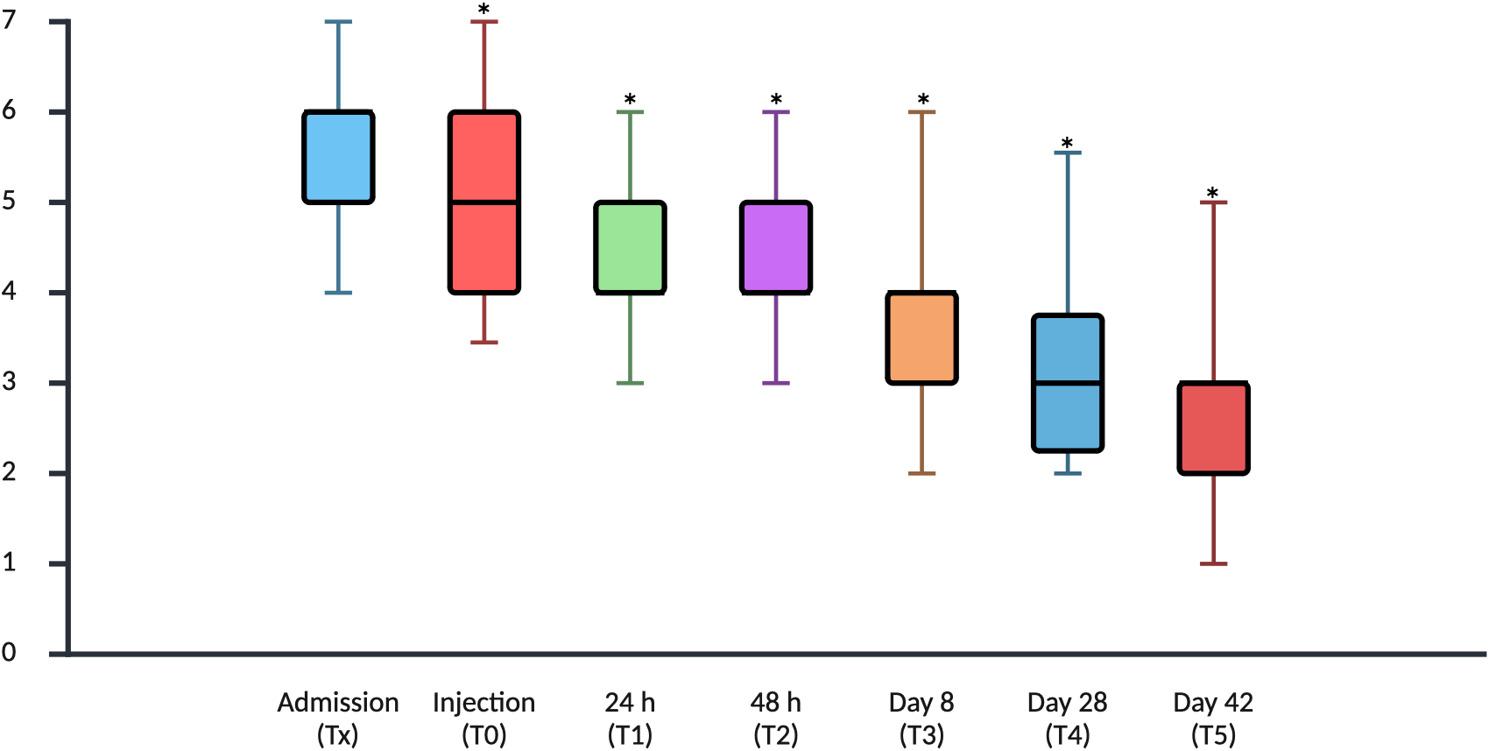
CGI-BP severity over time. Boxplots showing Clinical Global Impression for Bipolar Disorder—Severity (CGI-BP-S; overall anchor; 1 = normal, not at all ill; 7 = among the most extremely ill patients) at Tx, T0 (first risperidone ISM injection following ≥6 days of oral risperidone lead-in), T1, T2, T3, T4 and T5. Boxes represent the interquartile range with the median line; whiskers indicate dispersion. * indicates statistically significant reductions versus baseline (Tx) using Wilcoxon signed-rank tests with Holm–Bonferroni correction (p<0.05)

Global clinical severity, as measured by the CGI-BP, improved in parallel with YMRS (Friedman *p* < 0.0001; Table 2; Fig. 2). The median CGI-BP at Tx was 6 [5–6], consistent with “markedly” to “severely” ill patients. Scores decreased to 5 [4–6] at T0 (initiation of once monthly Risperidone ISM), followed by 4 [4–5] at T1 and T2, and 4 [3–4] at T3. By T4, the median CGI-BP further improved to 3 [2–4], and remained 3 [2–3] at T5, indicating a shift towards “mildly” to “moderately” ill.

All post-baseline CGI-BP scores were significantly lower than Tx after Holm–Bonferroni correction, confirming a robust clinical impression of improvement (Fig. 2).

Consistent with YMRS, mixed-effects models estimated a progressive reduction in LS-mean CGI-BP severity scores from 5.7 at admission to 2.8 at day 42. All prespecified pairwise contrasts (Tx–T0, T0–T2, T2–T3, T3–T4) were statistically significant (all Holm-adjusted *p* < 0.001), supporting a steady improvement in global illness severity over the observation period (see Additional file 2 : Supplementary - Table S2*).*

Consistent with the YMRS findings, pairwise comparisons also indicated significant decreases in CGI-BP scores from T0 to subsequent early post-injection visits, particularly up to day 8 (Table 2).

#### Sensitivity analyses

Covariate-adjusted mixed models that included baseline age, sex, BMI, and concomitant lithium and valproate treatment yielded LS-mean trajectories for YMRS and CGI-BP that were virtually identical to those from the unadjusted models. For YMRS, all prespecified contrasts from admission to day 28 remained highly significant after Holm–Bonferroni correction, whereas the contrast between day 28 and day 42 remained non-significant, confirming rapid early improvement followed by stabilisation. Likewise, adjustment did not materially alter the magnitude or significance of the CGI-BP contrasts, which continued to show a consistent, progressive reduction in global illness severity (see Additional file 2: Supplementary - Tables S1C and S2C*).*

### YMRS single-item trajectories

**Fig. 3:**
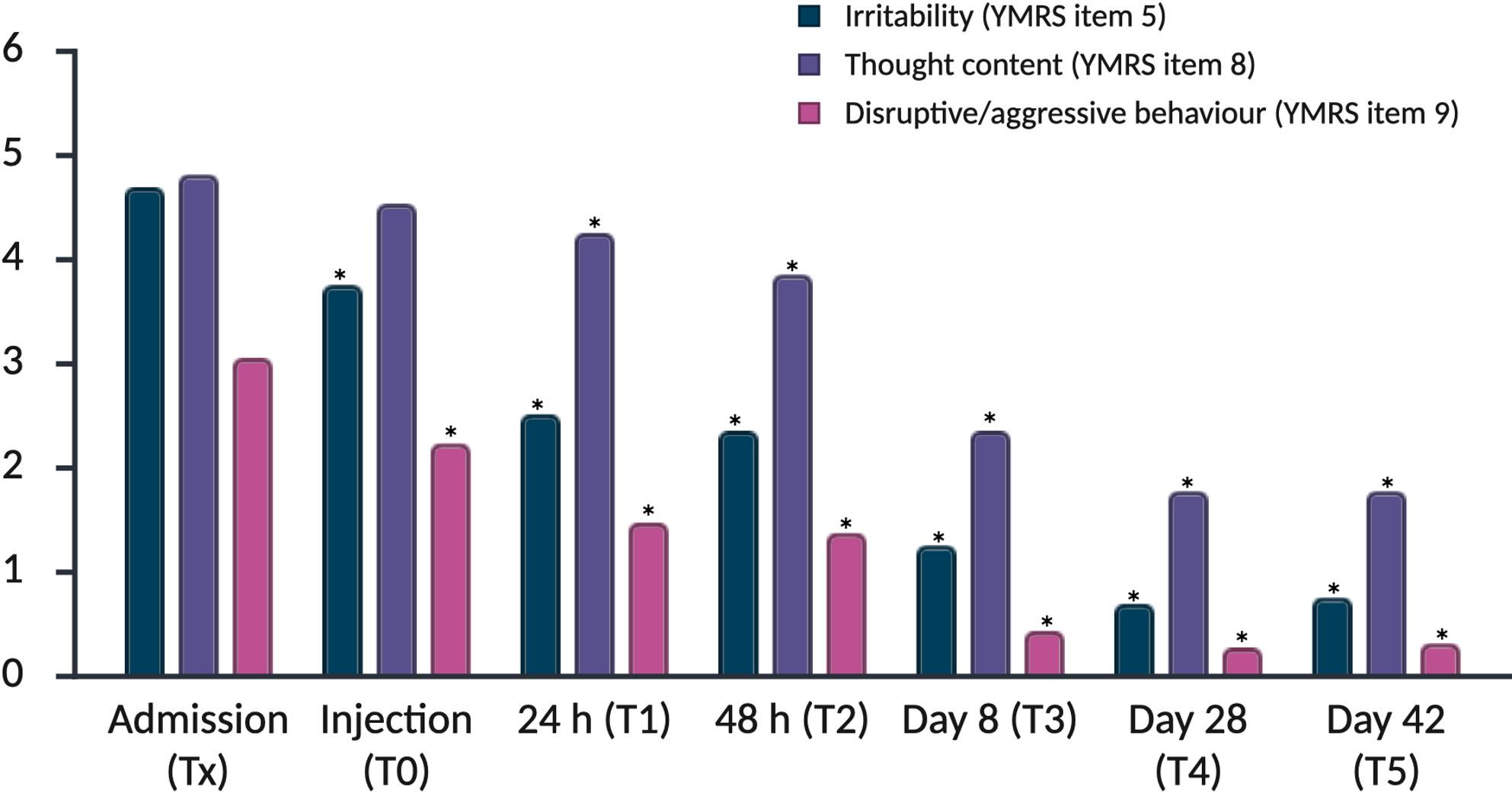
Trajectories of selected YMRS single items. Bar chart depicting median scores over time for three predefined YMRS items reflecting key symptom domains: irritability (item 5), thought content/psychotic ideation (item 8), and disruptive/aggressive behaviour (item 9). Time points are Tx, T0 (first risperidone ISM injection following ≥6 days of oral risperidone lead-in), T1, T2, T3, T4 and T5 as defined in the Methods. * indicates statistically significant differences versus baseline (Tx) in Wilcoxon signed-rank tests with Holm–Bonferroni correction (p<0.05)

Single-item YMRS trajectories were examined as exploratory analyses to descriptively characterize symptom change patterns over time. Changes in YMRS single items across all time points are summarised and depicted in Fig. 3.

Friedman tests indicated overall change over time for each item (all *p* < 0.0001), but item-level inferential findings were considered exploratory. Overall, YMRS single items decreased over time, with most of the decline occurring between Tx/T0 and T3.

As regard core YMRS single items (see Table 2; Fig. 3), “Irritability” showed one of the steepest early improvements, with medians falling from 5 [4–5] at Tx to 4 [3–5] at T0, 2 [2–3] at T1–T2, 1 [1–2] at T3, and 1 [0–1] at T4–T5.

“Aggressive behaviour” decreased from 2 [2–5] at Tx to 2 [1–2] at T0, 2 [1–2] at T1, and 1 [0–2] at T2. By T3, the median score was 0 [0–1], and 0 [0–0] at T4 and T5, indicating near-complete reduction of overt aggressive behaviours in most patients.

Psychotic “thought content” (Item 8) showed a more gradual trajectory. Median scores decreased from 5 [5–5] at Tx to 5 [4–5] at T0–T1, 4 [3–5] at T2, and only then to 2 [1–3] at T3, stabilising at 2 [1–2] at T4–T5. This pattern suggests that psychotic ideation tended to normalise more slowly, following the early antimanic and behavioural changes.

### Tolerability and side effects

GASS analyses were intentionally focused on clinically distressing side effects (as defined using the patient distress field and clinical review), and therefore do not reflect the overall frequency of all side effects. This distress-filtered GASS approach was intended as a pragmatic measure of patient-perceived side-effect burden during clinical stabilisation rather than a comprehensive tolerability/safety assessment; accordingly, the low median scores observed should not be interpreted as absence of side effects, and early acute-phase or non-distressing adverse effects may be under-captured.

Distress-filtered GASS burden at week 4 (T4) and week 6 (T5) was very low, with median values of 0 (IQR 0–0; range 0–7 at T4 and 0–4 at T5). These low values are consistent with the predefined focus on distressing symptoms rather than all possible side effects.

**Fig. 4:**
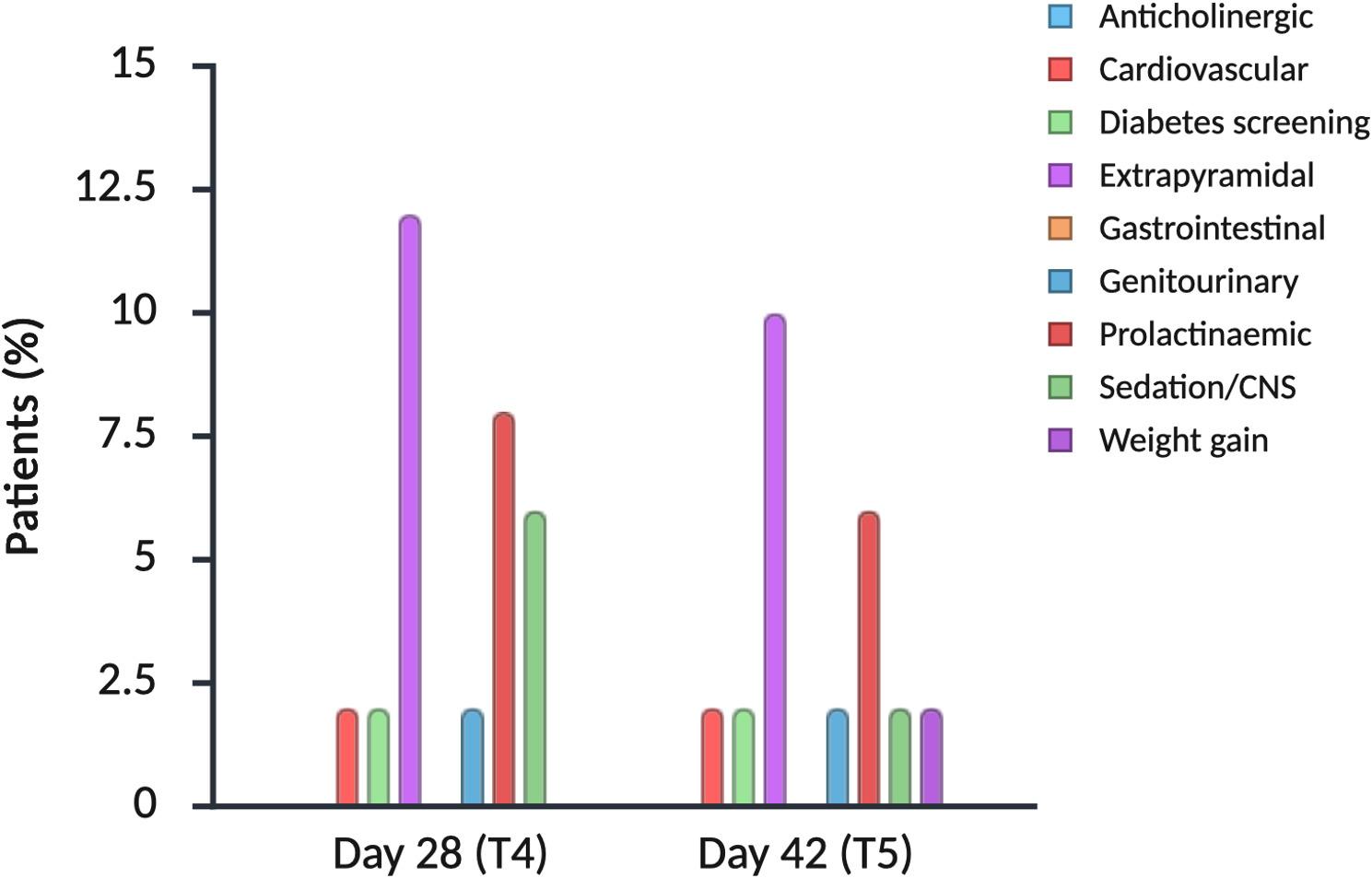
Prevalence of clinically distressing GASS domain symptoms at weeks 4 and 6. Grouped bar chart showing the proportion of patients reporting at least one clinically distressing symptom in each Glasgow Antipsychotic Side-Effect Scale (GASS) domain at day 28 ± 2 (T4) and day 42 ± 2 (T5). Clinically distressing symptoms were defined a priori using the patient distress field (distress ≥5/10 or “distressing” tick-box, plus clinical review), and non-distressing endorsed symptoms were not included in these prevalence summaries. Domains include sedation/CNS, cardiovascular, extrapyramidal, anticholinergic, gastrointestinal, genitourinary, prolactinaemic/sexual, diabetes-screening items, and weight gain. Percentages are calculated using N=50 at each time point

Analysis of GASS domains confirmed a low prevalence of clinically distressing side effects (Fig. 4). At T4, at least one clinically distressing symptom within the domain was reported by 3/50 patients (6.0%) in the sedation/CNS domain, 1/50 (2.0%) in the cardiovascular domain, 6/50 (12.0%) for extrapyramidal symptoms, 1/50 (2.0%) in the genitourinary domain, 1/50 (2.0%) in the diabetes-screening items and 4/50 (8.0%) for prolactinaemic/sexual symptoms; no patient reported clinically distressing anticholinergic or gastrointestinal symptoms, and no patient reported clinically distressing weight-gain symptoms at T4. At T5, domain prevalences were similar or slightly reduced: sedation/CNS symptoms in 1/50 (2.0%) patients, cardiovascular in 1/50 (2.0%), extrapyramidal in 5/50 (10.0%), genitourinary in 1/50 (2.0%), diabetes-screening items in 1/50 (2.0%), prolactinaemic/sexual in 3/50 (6.0%) and weight gain in 1/50 (2.0%), with anticholinergic/gastrointestinal symptoms remaining absent. Across domains, distress-filtered scores remained low, with no evidence of clinically significant distress burden.

No serious adverse events (AEs) were documented during the observation window. Importantly, no patient discontinued risperidone ISM because of tolerability issues during the observation period. Within the limits of a distress-focused assessment at weeks 4 and 6, no unexpected safety signals emerged.

## Discussion

To our knowledge, this is the first real-world study specifically examining once-monthly risperidone ISM in a cohort of non-adherent inpatients with schizoaffective disorder, bipolar type, admitted for an acute manic episode with psychotic symptoms and a documented previous response to risperidone. In this retrospective, uncontrolled cohort, we primarily describe symptom trajectories and short-term tolerability observed under a pragmatic inpatient pathway in which risperidone ISM was introduced after a short oral risperidone lead-in and within a multimodal treatment regimen. Accordingly, risperidone ISM should be viewed primarily as the continuity-enabling component of the pathway—supporting maintenance of antipsychotic exposure after acute stabilisation and potentially facilitating continuity beyond discharge—rather than as the sole driver of the short-term symptom improvement observed during inpatient care.

YMRS total scores decreased steeply over the early post-injection window and the vast majority of patients maintained improvement through week 6, with high proportions meeting standard response and remission thresholds by day 8 after the LAI injection and beyond. CGI-BP ratings closely mirrored the YMRS trajectory, indicating that symptom-scale change was reflected in global clinical impression. CGI-BP (*CGI-BP-S*,* overall anchor*) was included alongside YMRS as a complementary global measure, providing convergent clinician-rated information on overall severity in a manic episode with psychotic symptoms, whereas YMRS offers a more granular symptom-level assessment. The parallel improvement across YMRS and CGI-BP supports the internal consistency of the observed clinical course in this real-world inpatient pathway. It should be emphasized, however, that all patients received at least 6 consecutive days of oral risperidone before the first ISM injection, in addition to mood stabilizers and benzodiazepines in most cases and, in a minority, other antipsychotics. Thus, early symptomatic improvement likely reflects the combined effect of oral risperidone, risperidone ISM and concomitant treatments, rather than the LAI formulation alone. Tolerability findings should be interpreted in light of the predefined focus on clinically distressing side effects assessed at weeks 4 and 6; within this distress-filtered approach, no unexpected safety signals emerged, whereas early acute-phase and/or non-distressing side effects may be under-captured.

These observations are consistent with the evidence supporting risperidone’s antimanic efficacy that has been demonstrated in randomized and open-label studies in bipolar I disorder and related mood–psychotic conditions, including long-acting risperidone formulations [12–15]. They also align with pharmacokinetic and clinical trial data in schizophrenia showing early therapeutic exposure and sustained delivery with monthly risperidone ISM [17, 18, 24]. Risperidone ISM employs an in situ microimplant technology in which, after reconstitution and intramuscular injection, a proportion of risperidone is released early while the remainder precipitates into a polymeric matrix that biodegrades over the dosing interval [18]. The temporal pattern observed in our cohort—early antimanic improvement followed by maintenance of low YMRS scores at later visits—may be compatible with this biphasic release profile, although causal attribution is not possible and the respective contributions of oral lead-in, ISM, co-interventions, and inpatient environmental factors cannot be disentangled.

A distinctive aspect of this study is the evaluation of YMRS single-item trajectories. Activation-related and behavioral dimensions (e.g., irritability, disruptive/aggressive behavior, etc.) showed steep early reductions, reaching very low median values by day 8. In contrast, thought content (reflecting psychotic and cognitive aspects of schizoaffective disorder) improved more gradually. This temporal dissociation is clinically plausible: motor activation, irritability and overt aggression are often the earliest targets of pharmacological and environmental containment on acute wards, whereas psychotic ideation and insight may normalize more slowly. The present data quantify this pattern in a diagnostically homogeneous schizoaffective sample and suggest that a monthly LAI strategy incorporating risperidone ISM may be particularly useful when rapid behavioral stabilization is required in patients with high risk of non-adherence. However, these patterns should be regarded as descriptive and hypothesis-generating within this care pathway.

From a clinical standpoint, these results have several implications. First, in schizoaffective patients with non-adherence and prior response to risperidone, a combined oral lead-in and transition to once-monthly risperidone ISM may represent a pragmatic strategy to re-establish therapeutic antipsychotic exposure and secure continuity of treatment beyond the acute phase—consistent with guideline recommendations and evidence supporting LAIs in patients at high risk of non-adherence and relapse [8, 10, 11, 25–27]. Second, the rapid improvement in behavioral dimensions coupled with the slower but robust change in psychotic thought content supports integration of LAI strategies into multimodal management (including mood stabilizers, benzodiazepines when indicated, and psychosocial interventions) for complex manic presentations with psychosis. Third, the low post-acute YMRS levels observed by day 8 raise the hypothesis that, in selected patients, earlier discharge with continued LAI treatment and structured community follow-up might be feasible—a hypothesis that warrants prospective evaluation and should not be interpreted as a recommendation based on efficacy of ISM per se [27].

### Strengths and limitations

This study has several strengths. The sample is diagnostically homogeneous, focusing specifically on DSM-5-TR schizoaffective disorder, bipolar type, in a manic episode with psychotic features [1]. The design is naturalistic and reflects routine practice in an acute psychiatric unit, where comorbidity and polypharmacy are common and non-adherence is a frequent precipitant of admission [4–6]. Multiple time points were available, including very early assessments and follow-up to six weeks, allowing granular description of both global and item-level trajectories. The use of the GASS provided a structured, patient-centered assessment of antipsychotic side effects at two clinically relevant follow-up visits [23].

Important limitations must also be acknowledged. First, the retrospective, uncontrolled design precludes firm causal inference about the specific contribution of risperidone ISM; outcomes reflect a combined inpatient strategy rather than the effect of the LAI formulation in isolation. Symptom improvement may be partly attributable to the natural course of acute mania, regression to the mean, restoration of sleep, structured containment and milieu effects, and concomitant treatments. Because this was a retrospective, naturalistic inpatient cohort, concomitant treatments (mood stabilisers, benzodiazepines and PRN medications) were adjusted according to clinical judgement and behavioural containment needs, often on a day-by-day basis. Although granular reconstruction of scheduled and PRN dosing over time was not feasible due to retrospective documentation variability, chart review suggested a progressive tapering of benzodiazepines after the acute phase and maintenance rather than systematic escalation of mood stabilizers during the post-injection follow-up. Residual confounding from co-interventions and variable adherence to any oral medication in this clinical population cannot be excluded; adjusted models should be interpreted as limited sensitivity analyses rather than as fully addressing confounding. Accordingly, any apparent ‘effect’ is inseparable from concomitant treatments, structured containment, and the natural course of acute mania.

Second, all patients underwent at least 6 days of oral risperidone before the first ISM injection; the separate impact of the oral lead-in versus the LAI component was not formally modeled. Third, the sample size is modest and recruitment was from a single center, limiting generalizability. Fourth, because inclusion required a documented prior clinical response to risperidone, this cohort represents an enriched responder population, which may inflate apparent treatment responsiveness and limits generalisability; findings should not be extrapolated to risperidone-naïve patients or individuals with documented non-response to risperidone. Fifth, ratings were performed in routine practice without formal inter-rater reliability assessment, and documentation bias cannot be excluded. Moreover, YMRS and CGI-BP are rater-sensitive measures and ratings were performed by treating clinicians in routine care without independent raters or formal inter-rater reliability checks; expectancy/observer bias cannot be excluded. Item-level findings should be considered hypothesis-generating and interpreted cautiously due to multiple comparisons.

Sixth, tolerability was evaluated only over six weeks; metabolic, endocrine and cardiovascular effects beyond this window were not systematically captured and may be under-detected early after acute hospitalization. Tolerability summaries were intentionally focused on clinically distressing side effects (distress-filtered GASS approach) at weeks 4 and 6; therefore, these results should not be interpreted as the overall frequency of side effects, and early acute-phase and/or non-distressing adverse effects may be under-captured. Objective laboratory monitoring (e.g., prolactin and metabolic parameters) - which could contribute to define tolerability - was not systematically available in this retrospective dataset, because investigations beyond routine baseline assessment were requested only when clinically indicated in usual care; therefore, such outcomes could not be reported comprehensively. Finally, use of risperidone ISM in acute schizoaffective mania is off-label, underscoring the exploratory nature of these findings and the need for prospective controlled studies.

## Conclusions

In a real-world cohort of non-adherent inpatients with schizoaffective disorder, bipolar type, hospitalized for acute mania with psychotic symptoms and prior response to risperidone, a pragmatic strategy consisting of a brief oral risperidone lead-in followed by initiation of once-monthly risperidone ISM within a multimodal regimen was associated with rapid and marked reductions in manic symptom severity, progressive improvement in psychotic thought content, and a favorable short-term tolerability profile. In this uncontrolled inpatient cohort, risperidone ISM should be interpreted primarily as a continuity-enabling component of the pathway—supporting maintenance of antipsychotic exposure beyond acute stabilisation—rather than as a standalone causal driver of symptom improvement. Short-term tolerability, assessed at weeks 4 and 6 with a distress-filtered patient-reported approach, showed a low burden of clinically distressing side effects; early acute-phase and/or non-distressing adverse effects may be under-captured.

Activation-related and behavioral symptoms improved particularly quickly, whereas psychotic thought content normalized more gradually but substantially over the 6-week observation period. The overall temporal pattern observed under this pathway may be compatible with the formulation characteristics of risperidone ISM (early release with sustained delivery across the monthly dosing interval) [17]. However, the present findings cannot disentangle the respective contributions of the oral lead-in, ISM, concomitant medications, and structured inpatient care (including sleep restoration and containment), and therefore should not be interpreted as evidence of ISM efficacy per se. Use of risperidone ISM in this acute schizoaffective manic presentation is off-label, and clinical implications should remain conservative and hypothesis-generating.

Future prospective, randomized and ideally pragmatic trials—comparing risperidone ISM with oral antipsychotics and other LAIs and incorporating longer-term safety, functional and quality-of-life outcomes—are warranted to confirm these preliminary observations and to better define the role of ISM formulations in severe mood–psychotic disorders [10, 11, 25, 27].

## Supplementary Information

Supplementary Material 1

Supplementary Material 2

## Data Availability

The data generated and/or analysed during the current study are not publicly available due to ethical and legal restrictions related to patient confidentiality, but de-identified data may be made available from the corresponding author upon reasonable request and subject to Ethics Committee approval.
