# Supplementary material for: Risperidone long-acting in-situ microimplant following a brief oral risperidone lead-in in the acute inpatient management of manic episodes with psychotic symptoms in non-adherent patients with schizoaffective disorder: a retrospective, uncontrolled real-world study
Source: Ann Gen Psychiatry. 2026 May 28;25:64. doi: 10.1186/s12991-026-00674-1 (PMC13403579; doi:10.1186/s12991-026-00674-1)
Supplement: Supplementary file 1 — Supplementary Material 1 [file 12991_2026_674_MOESM1_ESM.doc]

**STROBE Statement—Checklist**

| ***Section*** | ***Item No.*** | ***Recommendation*** | ***Reported on*** |
| --- | --- | --- | --- |
| **TITLE AND ABSTRACT** |  |  |  |
|  | 1a | Indicate the study’s design with a commonly used term in the title or the abstract | Title; Abstract (Methods): p1 l1–3; p1 l22–25 |
|  | 1b | Provide in the abstract an informative and balanced summary of what was done and what was found | Abstract: p1 l13–25; p2 l26–36 |
| **INTRODUCTION** |  |  |  |
|  | 2 | Explain the scientific background and rationale for the investigation being reported | 1. Introduction: p2 l42–66; p3 l67–83; p4 l84–87 |
|  | 3 | State specific objectives, including any prespecified hypotheses | End of 1. Introduction (final paragraph: “This retrospective study was therefore undertaken…”): p4 l88–92 |
| **METHODS** |  |  |  |
|  | 4 | Present key elements of study design early in the paper | 2.2 Study design and setting: p5 l103–115 |
|  | 5 | Describe the setting, locations, and relevant dates, including periods of recruitment, exposure, follow-up, and data collection | 2.2; 2.2.2.1 (time points); study period Jan 2024–Nov 2025: p5 l104–110; p7 l170–180 |
|  | 6a | Give the eligibility criteria, and the sources and methods of selection of participants. Describe methods of follow-up | 2.2.1 Inclusion; 2.2.2 Exclusion; 2.2.2.1 (Tx–T5): p5 l104–110; p5 l117–127; p6 l129–149; p7 l170–180 |
|  | 6b | For matched studies, give matching criteria and number of exposed and unexposed | N/A (single cohort; no matching) |
|  | 7 | Clearly define all outcomes, exposures, predictors, potential confounders, and effect modifiers. Give diagnostic criteria, if applicable | 2.2 (DSM-5-TR); 2.2.1–2.3.1; Table 1: p5 l107–109; p5 l122–126; p7 l158–169; p11 l265–284; p13 l316–320 |
|  | 8* | For each variable of interest, give sources of data and details of methods of assessment (measurement). Describe comparability of assessment methods if there is more than one group | 2.2.2.1 Data sources/measurements; assessment time points; rating scales; GASS; AEs: p7 l158–169; p7 l170–180; p8 l185–206; p9 l207–231; p10 l232–235 |
|  | 9 | Describe any efforts to address potential sources of bias | 4. Discussion—Strengths and limitations; 2.4 (sensitivity/adjusted models): p8 l181–184; p13 l315–320; p27 l619–634; p28 l647–656 |
|  | 10 | Explain how the study size was arrived at | 2.2 (consecutive admissions); 3.1 (66 screened → 50 included): p5 l105–110; p14 l346–356 |
|  | 11 | Explain how quantitative variables were handled in the analyses. If applicable, describe which groupings were chosen and why | 2.4; 2.3.1 (response/remission thresholds; GASS categories): p5 l122–124; p9 l213–222; p11 l278–279; p12 l287–308; p13 l330–338 |
|  | 12a | Describe all statistical methods, including those used to control for confounding | 2.4 Statistical analysis (Friedman/Wilcoxon+Holm; mixed models; adjusted models): p12 l287–308; p13 l309–327; p13 l315–320; p13 l330–338 |
|  | 12b | Describe any methods used to examine subgroups and interactions | N/A (not performed / not prespecified). Exploratory baseline correlates referenced: p19 l435–436 (Additional file 2, Table S3) |
|  | 12c | Explain how missing data were addressed | 3.1 (complete assessments; GASS available for all); 2.4 (complete-case implied): p9 l230–231; p13 l328–329; p14 l354–355 |
|  | 12d | If applicable, explain how loss to follow-up was addressed | N/A (retrospective cohort; complete planned assessments): p14 l353–355 |
|  | 12e | Describe any sensitivity analyses | 2.4 (adjusted mixed models); 3.2.2 Sensitivity analyses: p13 l315–320; p21 l464–472 |
| **RESULTS** |  |  |  |
|  | 13a* | Report numbers of individuals at each stage of study—eg numbers potentially eligible, examined for eligibility, confirmed eligible, included in the study, completing follow-up, and analysed | 3.1 Cohort description: p14 l346–356 |
|  | 13b | Give reasons for non-participation at each stage | 3.1 (excluded due to incomplete documentation; substance-induced): p14 l347–352 |
|  | 13c | Consider use of a flow diagram | Not included; counts reported: p14 l346–356 |
|  | 14a* | Give characteristics of study participants (eg demographic, clinical, social) and information on exposures and potential confounders | Table 1; 3.1: p15 l358–359 (Table 1); p16 l360–379; p16 l380–382 |
|  | 14b | Indicate number of participants with missing data for each variable of interest | 3.1 (all assessments complete); Tables 1–2 (N=50): p13 l328–329; p14 l354–355 |
|  | 14c | Summarise follow-up time (eg, average and total amount) | 2.2.2.1 (Tx–T5 up to day 42); 3.1: p7 l170–180; p18 l391–392 |
|  | 15* | Report numbers of outcome events or summary measures over time | Table 2; Figures 1–4; 3.2–3.4: p17 l384–388 (Table 2); p18 l390–405; p19 l413–436; p20 l438–462; p21 l473–488; p22 l489–499; p22 l500–510; p23 l523–538 |
|  | 16a | Give unadjusted estimates and, if applicable, confounder-adjusted estimates and their precision (eg, 95% confidence interval). Make clear which confounders were adjusted for and why they were included | Table 2; 3.2–3.2.2; Additional file 2 (Tables S1/S2/S1C/S2C): p17 l384–388; p19 l422–425; p20 l456–460; p21 l464–472 |
|  | 16b | Report category boundaries when continuous variables were categorized | 2.3.1 (response ≥50%, remission YMRS<8); GASS categories; Table 1 substance categories: p5 l122–124; p11 l278–279; p9 l213–222; p7 l161–163 |
|  | 16c | If relevant, consider translating estimates of relative risk into absolute risk for a meaningful time period | N/A (no relative-risk estimates) |
|  | 17 | Report other analyses done—eg analyses of subgroups and interactions, and sensitivity analyses | 3.3 YMRS items; 3.2.2 sensitivity; Additional file 2 (Supplementary Table S3 predictors): p12 l305–308; p19 l435–436; p21 l464–472; p21–22 l473–499 |
| **DISCUSSION** |  |  |  |
|  | 18 | Summarise key results with reference to study objectives | 4. Discussion (opening paragraphs): p24 l540–551; p25 l552–568 |
|  | 19 | Discuss limitations of the study, taking into account sources of potential bias or imprecision. Discuss both direction and magnitude of any potential bias | 4. Discussion—Strengths and limitations: p27 l619–634; p28 l636–657 |
|  | 20 | Give a cautious overall interpretation of results considering objectives, limitations, multiplicity of analyses, results from similar studies, and other relevant evidence | 4. Discussion (interpretation + causal caveat): p24 l540–550; p25 l560–568; p25–26 l570–593; p26 l595–607 |
|  | 21 | Discuss the generalisability (external validity) of the study results | 4. Discussion—Strengths and limitations (single-centre; modest N): p28 l638–640 |
| **OTHER INFORMATION** |  |  |  |
|  | 22 | Give the source of funding and the role of the funders for the present study and, if applicable, for the original study on which the present article is based | Declarations—Funding: p31 l721–723 |
| **FOOTNOTE** |  |  |  |
|  | * | If applicable, give information separately for exposed and unexposed groups. Here: N/A (single cohort/no comparator). | N/A (single cohort/no comparator) |

FOOTNOTE

* If applicable, give information separately for exposed and unexposed groups. Here: N/A (single cohort/no comparator).

Information on the STROBE Initiative is available at http://www.strobe-statement.org.
