# Supplementary material for: Risperidone long-acting in-situ microimplant following a brief oral risperidone lead-in in the acute inpatient management of manic episodes with psychotic symptoms in non-adherent patients with schizoaffective disorder: a retrospective, uncontrolled real-world study
Source: Ann Gen Psychiatry. 2026 May 28;25:64. doi: 10.1186/s12991-026-00674-1 (PMC13403579; doi:10.1186/s12991-026-00674-1)
Supplement: Supplementary file 2 — Supplementary Material 2 [file 12991_2026_674_MOESM2_ESM.docx]

Supplementary

*P-values < 0.0001 are reported as ‘< 0.0001’; all other p-values are rounded to three decimal places.*

**Supplementary Table S1A. LS-means for YMRS total score by timepoint (unadjusted mixed model)**

Linear mixed-effects model with time (Tx, T0, T1, T2, T3, T4, T5) as categorical fixed effect (Tx reference) and random intercept for patient; REML estimation.

| Time | LS-mean YMRS | SE | 95% CI lower | 95% CI upper |
| --- | --- | --- | --- | --- |
| Tx | 31.98 | 0.85 | 30.31 | 33.65 |
| T0 | 26.58 | 0.85 | 24.91 | 28.25 |
| T1 | 21.56 | 0.85 | 19.89 | 23.23 |
| T2 | 19.00 | 0.85 | 17.33 | 20.67 |
| T3 | 8.74 | 0.85 | 7.07 | 10.41 |
| T4 | 5.90 | 0.85 | 4.23 | 7.57 |
| T5 | 6.18 | 0.85 | 4.51 | 7.85 |

**Supplementary Table S1B. Prespecified pairwise contrasts of LS-means for YMRS total score (unadjusted mixed model)**

Same model as in S1A. Contrasts are B−A (later time minus earlier time). Wald tests with normal approximation; Holm–Bonferroni adjustment across the six contrasts.

| Contrast | LS-mean difference (B−A) | SE | 95% CI lower | 95% CI upper | p-value | Holm-adjusted p-value |
| --- | --- | --- | --- | --- | --- | --- |
| Tx vs T0 | −5.40 | 0.90 | −7.16 | −3.64 | < 0.0001 | < 0.0001 |
| T0 vs T1 | −5.02 | 0.90 | −6.78 | −3.26 | < 0.0001 | < 0.0001 |
| T0 vs T2 | −7.58 | 0.90 | −9.34 | −5.82 | < 0.0001 | < 0.0001 |
| T2 vs T3 | −10.26 | 0.90 | −12.02 | −8.50 | < 0.0001 | < 0.0001 |
| T3 vs T4 | −2.84 | 0.90 | −4.60 | −1.08 | 0.002 | 0.003 |
| T4 vs T5 | 0.28 | 0.90 | −1.48 | 2.04 | 0.755 | 0.755 |

**Supplementary Table S1C. Covariate-adjusted LS-means and prespecified contrasts for YMRS total score (sensitivity analysis)**

Linear mixed-effects model with time (Tx, T0, T1, T2, T3, T4, T5) as categorical fixed effect (Tx reference), baseline age, sex (SEX), BMI, lithium use (LITHIUM_any) and any valproate use (VALPROATE_any) as additional fixed effects, and random intercept for patient; REML estimation. LS-means evaluated at the sample means of covariates.
Covariate adjustment did not materially change the estimated YMRS trajectories; covariate-adjusted LS-means and prespecified contrasts were identical to the unadjusted estimates within rounding.

*S1C – Part 1. Covariate-adjusted LS-means for YMRS total score by timepoint*

| Time | LS-mean YMRS | SE | 95% CI lower | 95% CI upper |
| --- | --- | --- | --- | --- |
| Tx | 31.98 | 0.85 | 30.31 | 33.65 |
| T0 | 26.58 | 0.85 | 24.91 | 28.25 |
| T1 | 21.56 | 0.85 | 19.89 | 23.23 |
| T2 | 19.00 | 0.85 | 17.33 | 20.67 |
| T3 | 8.74 | 0.85 | 7.07 | 10.41 |
| T4 | 5.90 | 0.85 | 4.23 | 7.57 |
| T5 | 6.18 | 0.85 | 4.51 | 7.85 |

*S1C – Part 2. Covariate-adjusted prespecified pairwise contrasts of LS-means for YMRS total score*

| Contrast | LS-mean difference (B−A) | SE | 95% CI lower | 95% CI upper | p-value | Holm-adjusted p-value |
| --- | --- | --- | --- | --- | --- | --- |
| Tx vs T0 | −5.40 | 0.90 | −7.16 | −3.64 | < 0.0001 | < 0.0001 |
| T0 vs T1 | −5.02 | 0.90 | −6.78 | −3.26 | < 0.0001 | < 0.0001 |
| T0 vs T2 | −7.58 | 0.90 | −9.34 | −5.82 | < 0.0001 | < 0.0001 |
| T2 vs T3 | −10.26 | 0.90 | −12.02 | −8.50 | < 0.0001 | < 0.0001 |
| T3 vs T4 | −2.84 | 0.90 | −4.60 | −1.08 | 0.002 | 0.003 |
| T4 vs T5 | 0.28 | 0.90 | −1.48 | 2.04 | 0.755 | 0.755 |

**Supplementary Table S2A. LS-means for CGI-BP severity score by timepoint (unadjusted mixed model)**

*Linear mixed-effects model with time (Tx, T0, T1, T2, T3, T4, T5) as categorical fixed effect (Tx reference) and random intercept for patient; REML estimation.*

| Time | LS-mean CGI-BP | SE | 95% CI lower | 95% CI upper |
| --- | --- | --- | --- | --- |
| Tx | 5.70 | 0.15 | 5.40 | 6.00 |
| T0 | 5.12 | 0.15 | 4.82 | 5.42 |
| T1 | 4.54 | 0.15 | 4.24 | 4.84 |
| T2 | 4.26 | 0.15 | 3.96 | 4.56 |
| T3 | 3.66 | 0.15 | 3.36 | 3.96 |
| T4 | 3.10 | 0.15 | 2.80 | 3.40 |
| T5 | 2.84 | 0.15 | 2.54 | 3.14 |

**Supplementary Table S2B. Prespecified pairwise contrasts of LS-means for CGI-BP severity score (unadjusted mixed model)**

*Same model as in S2A. Contrasts are B−A (later time minus earlier time). Wald tests with normal approximation; Holm–Bonferroni adjustment across the four contrasts.*

| Contrast | LS-mean difference (B−A) | SE | 95% CI lower | 95% CI upper | p-value | Holm-adjusted p-value |
| --- | --- | --- | --- | --- | --- | --- |
| Tx vs T0 | −0.58 | 0.12 | −0.82 | −0.34 | < 0.0001 | < 0.0001 |
| T0 vs T2 | −0.86 | 0.12 | −1.10 | −0.62 | < 0.0001 | < 0.0001 |
| T2 vs T3 | −0.60 | 0.12 | −0.84 | −0.36 | < 0.0001 | < 0.0001 |
| T3 vs T4 | −0.56 | 0.12 | −0.80 | −0.32 | < 0.0001 | < 0.0001 |

**Supplementary Table S2C. Covariate-adjusted LS-means and prespecified contrasts for CGI-BP severity score (sensitivity analysis)**

Linear mixed-effects model with time (Tx, T0, T1, T2, T3, T4, T5) as categorical fixed effect (Tx reference), baseline age, sex (SEX), BMI, any lithium use (LITHIUM_any) and any valproate use (VALPROATE_any) as additional fixed effects, and random intercept for patient; REML estimation. LS-means evaluated at the sample means of covariates.
Covariate adjustment did not materially change the estimated CGI-BP trajectories; covariate-adjusted LS-means and prespecified contrasts were identical to the unadjusted estimates within rounding.

***S2C – Part 1. Covariate-adjusted LS-means for CGI-BP severity score by timepoint***

| Time | LS-mean CGI-BP | SE | 95% CI lower | 95% CI upper |
| --- | --- | --- | --- | --- |
| Tx | 5.70 | 0.15 | 5.40 | 6.00 |
| T0 | 5.12 | 0.15 | 4.82 | 5.42 |
| T1 | 4.54 | 0.15 | 4.24 | 4.84 |
| T2 | 4.26 | 0.15 | 3.96 | 4.56 |
| T3 | 3.66 | 0.15 | 3.36 | 3.96 |
| T4 | 3.10 | 0.15 | 2.80 | 3.40 |
| T5 | 2.84 | 0.15 | 2.54 | 3.14 |

*S2C – Part 2. Covariate-adjusted prespecified pairwise contrasts of LS-means for CGI-BP severity score*

| Contrast | LS-mean difference (B−A) | SE | 95% CI lower | 95% CI upper | p-value | Holm-adjusted p-value |
| --- | --- | --- | --- | --- | --- | --- |
| Tx vs T0 | −0.58 | 0.12 | −0.82 | −0.34 | < 0.0001 | < 0.0001 |
| T0 vs T2 | −0.86 | 0.12 | −1.10 | −0.62 | < 0.0001 | < 0.0001 |
| T2 vs T3 | −0.60 | 0.12 | −0.84 | −0.36 | < 0.0001 | < 0.0001 |
| T3 vs T4 | −0.56 | 0.12 | −0.80 | −0.32 | < 0.0001 | < 0.0001 |

**Supplementary Table S3. Exploratory baseline correlates of day-8 outcomes (T3)**

Exploratory analyses of baseline correlates of early outcome at T3 (day 8). For each predictor, a separate model was fitted, adjusted for baseline YMRS total score (Tx), except for the baseline YMRS row (unadjusted). Remission at T3 was defined as YMRS<8. Linear models use robust (HC1) standard errors. Results are hypothesis-generating; no multiplicity correction was applied.

| Predictor (value OR n - %) | Remission at T3 (OR, 95% CI; p) (logistic, adj. for Tx YMRS) | YMRS total at T3 (β, 95% CI; p) (linear, adj. for Tx YMRS) | ΔYMRS Tx→T3 (β, 95% CI; p) (linear, adj. for Tx YMRS) |
| --- | --- | --- | --- |
| YMRS at admission (per 1 point) | 0.81 (0.71–0.92), **p=0.001** | +0.38 (+0.24–+0.52), **p<0.0001** | +0.62 (+0.48–+0.76), **p<0.0001** |
| Age (years) | 1.01 (0.96–1.06), p=0.759 | +0.00 (-0.05–+0.05), p=0.920 | -0.00 (-0.05–+0.05), p=0.920 |
| Sex (female vs male; 15/50, 30.0% female) | 0.91 (0.23–3.54), p=0.893 | -0.40 (-1.96–+1.16), p=0.614 | +0.40 (-1.16–+1.96), p=0.614 |
| BMI (kg/m^2) | 0.99 (0.90–1.09), p=0.863 | +0.01 (-0.09–+0.12), p=0.781 | -0.01 (-0.12–+0.09), p=0.781 |
| Substance use disorder (any vs none; 18/50 – 36%) | 0.87 (0.24–3.14), p=0.831 | +0.14 (-1.37–+1.66), p=0.852 | -0.14 (-1.66–+1.37), p=0.852 |
| Medical comorbidity (17/50 – 34%) | 0.28 (0.07–1.05), p=0.060 | +2.02 (+0.57–+3.48), **p=0.006** | -2.02 (-3.48–-0.57), **p=0.006** |
| Cluster B personality disorder (7/50 -14%) | 0.78 (0.15–4.01), p=0.766 | -0.16 (-1.77–+1.44), p=0.842 | +0.16 (-1.44–+1.77), p=0.842 |
| Lithium co-treatment (14/50-28%) | 4.74 (0.73–30.86), p=0.104 | -1.89 (-3.61–-0.17), **p=0.031** | +1.89 (+0.17–+3.61), **p=0.031** |
| Valproate co-treatment (33/50 – 66%) | 0.98 (0.26–3.66), p=0.979 | +0.17 (-1.25–+1.60), p=0.811 | -0.17 (-1.60–+1.25), p=0.811 |
| Risperidone ISM dose (100 mg= 41/50, 82%) | 0.14 (0.01–1.38), **p=0.092** | +0.22 (-1.77–+2.21), p=0.829 | -0.22 (-2.21–+1.77), p=0.829 |
| Duration of illness (years) | 1.00 (0.93–1.07), p=0.992 | +0.01 (-0.07–+0.08), p=0.840 | -0.01 (-0.08–+0.07), p=0.840 |
| Number of mood episodes | 1.04 (0.76–1.42), p=0.803 | -0.09 (-0.41–+0.23), p=0.575 | +0.09 (-0.23–+0.41), p=0.575 |

*Baseline YMRS severity* showed the strongest association with early outcome. After adjustment for baseline severity, medical comorbidity and lithium co-treatment showed exploratory associations with day-8 outcomes; estimates were imprecise and hypothesis-generating, and no multiplicity correction was applied.

Dose trajectories for co-treatments (lithium/valproate/benzodiazepines/antipsychotics) could not be extracted reliably due to retrospective documentation variability; therefore not reported.
